# Case report: Successful treatment of malignant pericardial effusion with pericardiocentesis, concurrent anti-inflammatory therapy and cancer therapy

**DOI:** 10.3389/fcvm.2023.1285233

**Published:** 2023-10-12

**Authors:** Nuri Lee, Hyunjin Bang, Hyukjin Park, Hyun-Jeong Shim

**Affiliations:** ^1^ Department of Cardiology, Chonnam National University Hwasun Hospital, Hwasun, Republic of Korea; ^2^ Department of Hematology and Oncology, Chonnam National University Medical School and Hwasun Hospital, Hwasun, Republic of Korea

**Keywords:** cardiac tamponade, constrictive pericarditis, pericardial effusion, breast cancer, pericardiocentesis

## Abstract

Despite significant advancements in systemic anticancer therapies, cardiac tamponade remains a serious and potentially life-threatening complication in metastatic breast cancer (MBC). However, there is a paucity of comprehensive research investigating alternative management approaches, such as pericardiocentesis and anti-inflammatory therapy (AIT), to effectively address cardiac tamponade and mitigate the risk of heart failure arising from constrictive physiology (CP) in patients with MBC when traditional systemic anticancer drugs fail to yield favorable outcomes. Herein, we describe two cases of MBC with cardiac tamponade that occurred despite the administration of effective systemic anticancer drugs. In each case, pericardial effusion was detected in a patient who was undergoing palliative anticancer therapy for human epidermal growth factor receptor 2 (HER2)-positive MBC. The patients in these cases were successfully treated with pericardiocentesis and AIT (prednisolone and colchicine) for subsequent CP without substitution with their systemic anticancer drugs. Cardiac tamponade and CP are regarded as signs of advanced cancer and are associated with a worse clinical outcome in general; however, they can still be treated with an effective anticancer drug, pericardiocentesis, and management of CP by cardiooncology specialists.

## Introduction

Metastatic breast cancer (MBC) infrequently involves the pericardium, and only a small proportion of patients with pericardial metastases present with cardiac tamponade (1, 2). Approximately half of pericardial effusion (PE) in patients with cancer is caused by the invasion of the primary malignancy into the pericardium; thus, PE is regarded as an independent predictor of poor prognosis (3, 4). Control of the underlying malignancy by systemic chemotherapy is thought to be the only way to improve clinical outcomes (5, 6); however, heart failure arising from constrictive physiology (CP) following pericardiocentesis frequently disturbs the success of chemotherapy. Moreover, recurrent PE requiring repeated pericardiocentesis can occur despite chemotherapy, which is effective against other metastases. This may be considered a progressive disease, that could permanently lead to discontinuation of current chemotherapy. However, there is a paucity of comprehensive research investigating alternative management approaches, such as pericardiocentesis and anti-inflammatory therapy (AIT), to effectively address cardiac tamponade and mitigate the risk of heart failure arising from CP in patients with MBC when traditional systemic anticancer drugs fail to yield favorable outcomes.

Herein, we present two cases in which overall control of MBC was ultimately achieved through pericardiocentesis and AIT for CP without switching to anticancer drugs despite the occurrence of malignant PE.

## Case description

### Case 1

**Figure 1:**
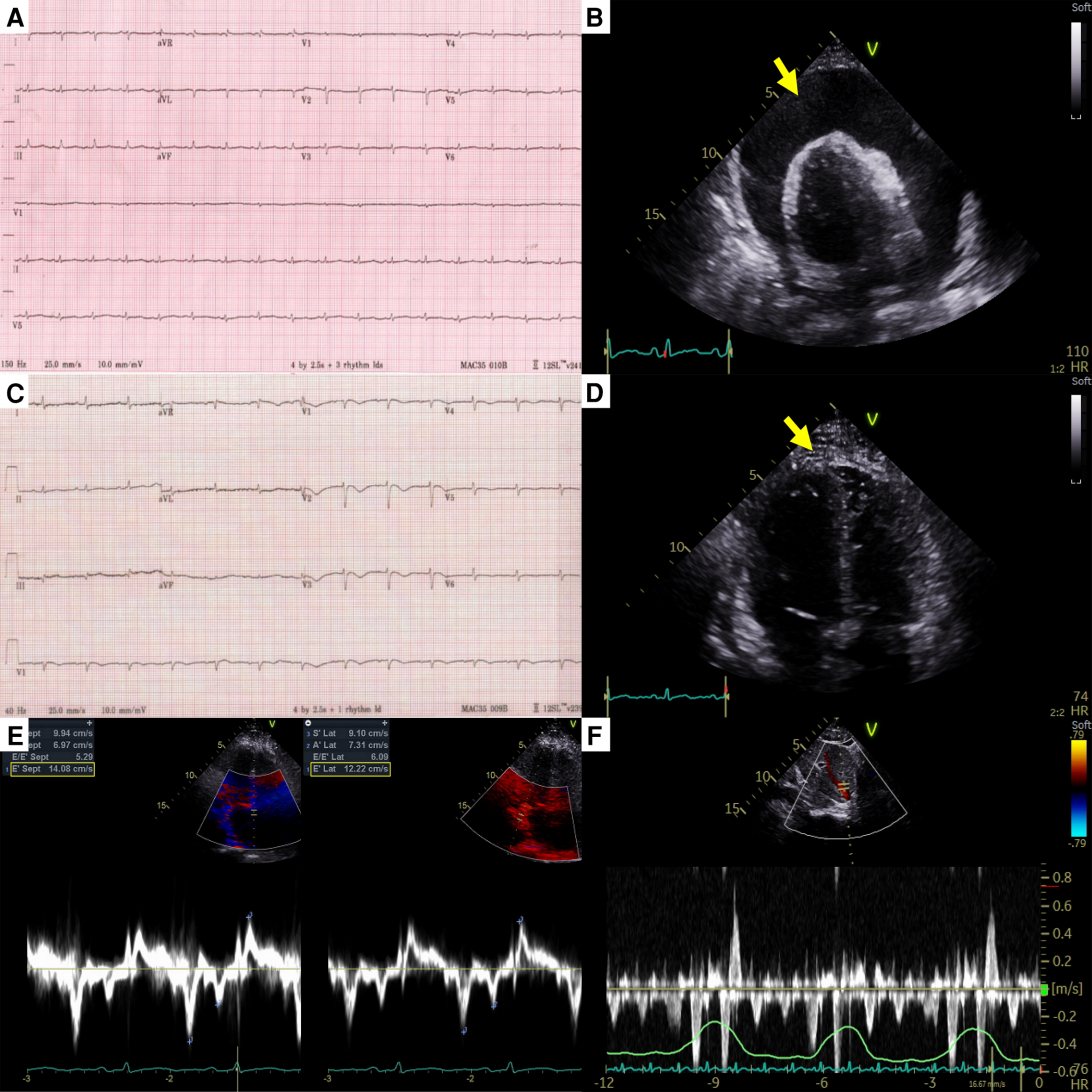
Electrocardiography and echocardiography at the first cardiac tamponade episode in case 1. (**A**) Low-voltage QRS complex in initial electrocardiography. (**B**) Large pericardial effusion (arrow) causing cardiac tamponade. (**C**) Newly developed anterior T wave inversion in post-pericardiocentesis electrocardiography. (**D**) Diffuse pericardial adhesions, and thickening (arrow) in post-pericardiocentesis echocardiography. (**E**) The medial early diastolic tissue doppler velocity exceeding the lateral velocity (annulus reversus). (**F**) Prominent expiratory diastolic flow reversal in the hepatic vein. Both (**E**) and (**F**) are suggestive of constrictive physiology.

**Figure 2:**
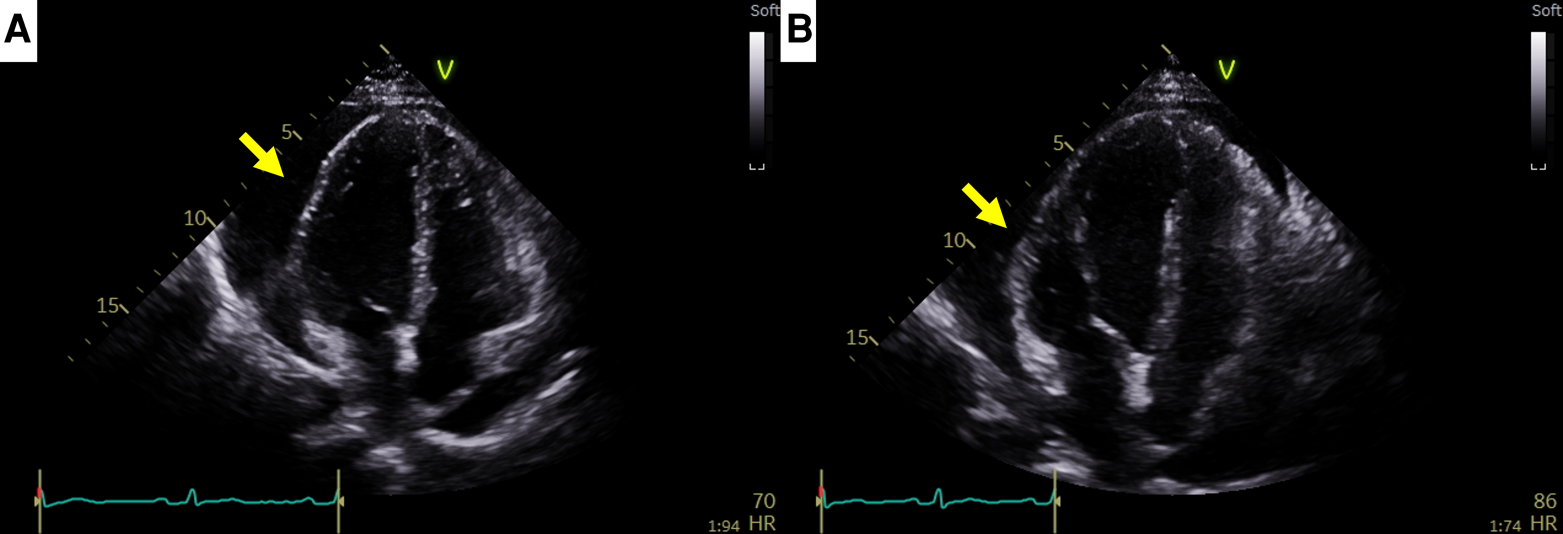
Recurrent cardiac tamponade episodes in case 1. (**A**) Second cardiac tamponade episode with large pericardial effusion (arrow). (**B**) Third cardiac tamponade episode with large pericardial effusion (arrow).

A 44-year-old female patient who had been undergoing 65 cycles of palliative chemotherapy with trastuzumab and pertuzumab for human epidermal growth factor receptor 2 (HER2)-positive MBC presented to the emergency department with dyspnea. The patient's blood pressure was 101/69 mmHg, and her pulse rate was slightly high at 105 beats per min (BPM). Initial electrocardiography (ECG) revealed a low-voltage QRS complex (Figure 1A), and her *N*-terminal pro-brain natriuretic peptide (NT-proBNP) level was relatively low (424 pg/ml). Therefore, we suspected cardiac tamponade and promptly performed echocardiography. A large PE with definite tamponade physiology was observed (Figure 1B, Supplementary Video 1), and emergent pericardiocentesis was performed. The next day, the ECG revealed a newly developed anterior T wave inversion (Figure 1C). Follow-up echocardiography showed markedly reduced PE, disseminated pericardial adhesions, and thickening with CP, suggesting effusive-constrictive pericarditis (ECP) (Figures 1D–F, Supplementary Video 2). Two days after pericardiocentesis, her dyspnea and tachycardia did not fully resolve, and the NT-proBNP level was elevated to 1,680 pg/ml, all of which were compatible with ECP. AIT against ECP was initiated using with prednisolone (started with 0.5 mg/kg/day, gradually tapered for 2 months) and colchicine (0.6 mg/day). A metastatic adenocarcinoma was identified on cytopathology in the drained PE, whereas no other possible causes of PE were identified in either serologic or PE analyses. Owing to the positive result of pericardial fluid cytopathology, we judged her breast cancer as a progressive disease and decided to change the cancer therapy to trastuzumab emtansine (T-DM1). However, cardiac tamponade recurred twice (2 and 5 months after initial pericardiocentesis, respectively), even after switching to T-DM1 (Figures 2A,B, Supplementary Videos 3 and 4), while other metastatic sites remained stable. Repeated pericardiocentesis and AIT for ECP were performed for each episode of cardiac tamponade. Despite recurrent cardiac tamponade episodes with T-DM1 therapy, we continued T-DM1 therapy with AIT because the symptoms and signs of heart failure were well controlled after pericardiocentesis and AIT. Furthermore, the oncological response to T-DM1 was thought to be stable, except for recurrent PE. Finally, after three times of pericardiocenteses, AIT, and eight cycles of T-DM1 therapy, cardiac tamponade has not recurred until now. The patient has been on T-DM1 maintenance therapy with an oncologic state of stable disease for two years.

### Case 2

**Figure 3:**
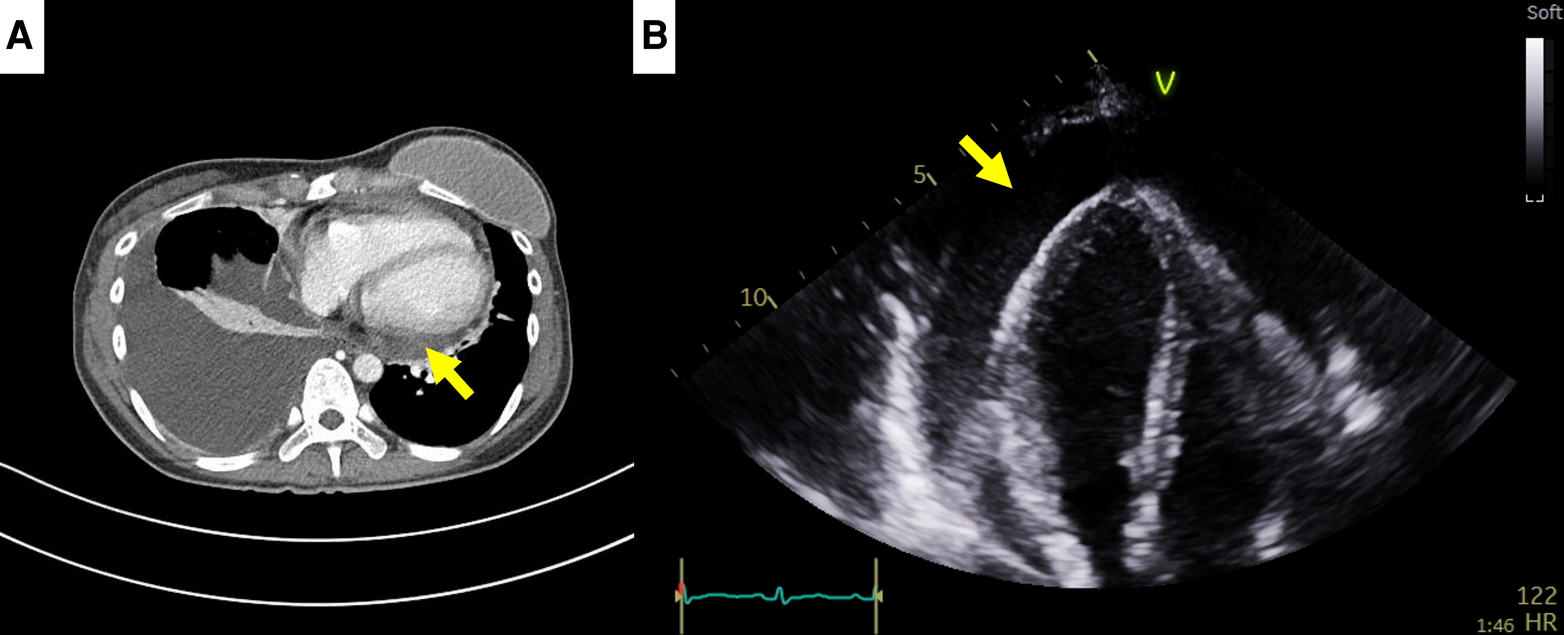
Computed tomography (CT) findings of case 2. (**A**) Small pericardial effusion in chest CT, 1 month prior to cardiac tamponade episode. (**B**) Large pericardial effusion (arrow) causing cardiac tamponade, 1 month after chest CT.

**Figure 4:**
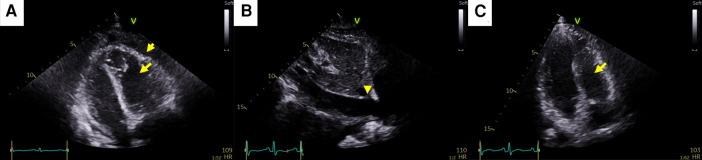
Pericardial changes after pericardiocentesis in case 2. (**A**) Pericardial adhesions and thickening (short arrow) causing constrictive physiology (CP). The right ventricular free wall was captured to the adjacent pericardium, which resulted in right ventricular systolic dysfunction (long arrow). (**B**) Plethora of the inferior vena cava caused by both CP and right ventricular systolic dysfunction (arrowhead). (**C**) At the end of anti-inflammatory treatment, pericardial adhesions, thickening, CP, and right ventricular dysfunction (arrow) were completely resolved.

A 35-year-old female patient who had been undergoing palliative chemotherapy with gemcitabine and cisplatin for HER2-positive MBC was referred to the Department of Cardiology for New York Heart Association (NYHA) class IV dyspnea. Her blood pressure was within the normal range (110/80 mmHg); however her pulse rate was elevated, reaching 124 BPM. The NT-proBNP level was slightly elevated (249 pg/ml). Echocardiography revealed a large PE with definite tamponade physiology, though only a small PE was detected on the chest computed tomography (CT) one month ago (Figures 3A,B, Supplementary Video 5). Emergent pericardiocentesis was performed on the same day, and a metastatic adenocarcinoma was identified in the PE cytopathology, without other possible causes of PE, similar to Case 1. Two days after the pericardiocentesis, her dyspnea only modestly improved (NYHA class III) and her NT-proBNP level was markedly elevated (4,230 pg/ml). Follow-up echocardiogram showed pericardial adhesions and thickening around the entire cardiac border with CP. Additionally, the right ventricular free wall was captured to the adjacent pericardium, which resulted in right ventricular systolic dysfunction (Figure 4A, Supplementary Video 6). Both CP and right ventricular systolic dysfunction resulted in plethora of the inferior vena cava (Figure 4B). AIT against ECP was also introduced for this patient using prednisolone and colchicine (the same regimen as in Case 1). Despite pericardial metastasis, chemotherapeutic regimen was maintained without changes, simultaneously with AIT because the overall cancer was relatively stable, except for pericardial metastasis. At the 14^th^ day of AIT, her dyspnea completely resolved (NYHA class I), and the NT-proBNP level rapidly decreased to 1,650 pg/ml. After 2 months of AIT, the NT-proBNP level decreased (245 pg/ml), and follow up echocardiogram showed complete resolution of pericardial thickening, adhesion, CP, and right ventricular dysfunction (Figure 4C, Supplementary Video 7). This patient is still receiving chemotherapy with gemcitabine and cisplatin for 9 months, without recurrence of CP, need for repeated pericardiocentesis, or cancer progression.

## Discussion

In these two cases, CP occurred after pericardiocentesis. While managing CP and heart failure with AIT, the current cancer therapy (T-DM1 and gemcitabine/cisplatin, respectively) were maintained. Little is known about adequate oncologic and cardiologic management of CP in patients with malignant PE. Despite ECP with or without recurrent cardiac tamponade events, we continued the current cancer therapy with AIT (and with repeated pericardiocentesis in Case 1) because CT imaging revealed a favorable response in other organs and tumor marker levels were not elevated. Eventually, we achieved an adequate oncologic response including the control of PE and ECP. These two cases give us a clinically important message: even if adverse pericardial events, such as ECP or recurrent cardiac tamponade, occur, proper AIT and/or repetitive pericardiocentesis can lead to the control of pericardial metastasis provided that the current anti-cancer drugs are effective in other organs.

The oncologic prognosis of malignant PE and concomitant cardiac tamponade associated with breast cancer has been known to be poor, with a median survival of 13 months (7). This might be caused not only by the heavy cancer burden represented by pericardial invasion, but also by difficulty in continuing cancer therapy due to heart failure symptoms associated with CP after pericardiocentesis. Patients with positive pericardial fluid cytopathologic results, especially when combined with CP, have lower event-free survival rates than those with negative cytopathological results (4, 5, 8). CP frequently develops after pericardiocentesis, and it is also associated with poor clinical outcomes in the patients by causing heart failure (9). Thus, patients who develop cardiac tamponade are often regarded as being in the terminal stage of cancer, especially when repetitive pericardiocentesis is required or when CP develops.

Several reports have documented successful treatment of MBC and cardiac tamponade, using thoracoscopic pericardial window surgery and chemotherapy (10, 11). However, in most cases, performing cardiac operations under general anesthesia in patients with advanced cancer is difficult because of their poor general condition and anesthetic risks. The pericardial window operation can cause postpericardiotomy syndrome, resulting in CP. Therefore, the pericardial window operation is not generalizable in real practice.

Recently, several studies have reported inflammatory and reversible CP after pericardiocentesis in patients with cancer (8, 9). From a therapeutic perspective, colchicine use after successful pericardiocentesis is associated with lower composite events (12). These results suggest that some patients may benefit from AIT by reducing pericardial inflammation and the consequent CP. Reduction in this process could relieve heart failure and improve the patient's general condition, enabling further cancer therapy against the primary malignancy.

## Conclusion

We report two cases of MBC with recurrent cardiac tamponade that were successfully treated with AIT and systemic anti-cancer therapy, with or without repetitive pericardiocentesis. Adverse pericardial events, such as ECP or recurrent cardiac tamponade, could be successfully managed using this treatment, in patients with malignant pericardial effusion. Long-term outcomes and prognostic indicators in these patients should be investigated, in further research.

## Data Availability

The original contributions presented in the study are included in the article/**Supplementary Material**, further inquiries can be directed to the corresponding author.

## Supplementary material

Supplementary Video 1 Large pericardial effusion causing cardiac tamponade in Case 1.

Supplementary Video 2 Diffuse pericardial adhesions, and thickening in post-pericardiocentesis echocardiography of Case 1.

Supplementary Video 3 Second cardiac tamponade episode with large pericardial effusion in Case 1.

Supplementary Video 4 Third cardiac tamponade episode with large pericardial effusion in Case 1.

Supplementary Video 5 Large pericardial effusion causing cardiac tamponade in Case 2.

Supplementary Video 6 Pericardial adhesions and thickening causing constrictive physiology. The right ventricular free wall was captured to the adjacent pericardium, which resulted in right ventricular systolic dysfunction.

Supplementary Video 7 At the end of anti-inflammatory treatment, pericardial adhesions, thickening, constrictive physiology, and right ventricular dysfunction were completely resolved.
